# Gly1057Asp polymorphism of insulin receptor substrate-2 is associated with coronary artery disease in the Taiwanese population

**DOI:** 10.1186/1423-0127-19-100

**Published:** 2012-12-05

**Authors:** Shih-Hung Chan, Jyh-Hong Chen, Yi-Heng Li, Liang-Miin Tsai

**Affiliations:** 1 Institute of Clinical Medicine, College of Medicine, National Cheng Kung University, Tainan, Taiwan; 2 Department of Internal Medicine, National Cheng Kung University Hospital, College of Medicine, National Cheng Kung University, Tainan, Taiwan

**Keywords:** Insulin receptor substrate, Diabetes mellitus, Coronary artery disease, Single nucleotide polymorphism, Insulin resistance, Homeostasis model assessment

## Abstract

**Background:**

Gly1057Asp polymorphism in insulin receptor substrate (IRS)-2 is related to insulin resistance and diabetes mellitus (DM), which both contribute to the pathogenesis of coronary artery disease (CAD). Hence, we hypothesize that Gly1057Asp polymorphism in IRS-2 is associated with CAD.

**Methods:**

Patients receiving elective coronary angiography were enrolled. Significant stenosis is defined as a luminal diameter stenosis greater than 50%. Patients without significant stenosis were defined as group A, and those with significant stenosis in at least one major coronary artery were defined as group B. Genotypes were determined by polymerase chain reaction/restriction fragment length polymorphism. Chi-square test and multivariate logistic regression were used to evaluate the relationship between Gly1057Asp polymorphism in IRS-2 and CAD. The homeostasis model assessment of insulin resistance (HOMA-IR) index was calculated as a representative of insulin resistance. Multiple linear regression was used to analyze the association between Gly1057Asp polymorphism in IRS-2 and the HOMA-IR index.

**Results:**

There were 170 patients in group A and 284 patients in group B. The Gly allele frequencies were 54.7% for group A and 60.9% for group B (p = 0.077). The Gly/Gly + Gly/Asp genotype frequency was 74.1% for group A and 84.9% for group B (p = 0.007). After adjustments for conventional risk factors in multivariate logistic regression, the odds ratio for CAD in patients with the Gly/Gly + Gly/Asp genotype was 2.008 [95% confidence interval (95% CI) = 1.210-3.332, p = 0.007], using patients with the Asp/Asp genotype as a reference group. The concurrence of Gly1057Asp polymorphism in IRS-2 with DM is correlated with occurrence of CAD. In multivariate logistic regression, employing non-diabetics with the Asp/Asp genotype as a reference group, the odds ratio for CAD was 1.561 [95% CI = 0.517-4.713, p = 0.430] for diabetics with the Asp/Asp genotype, 1.922 [95% CI = 1.086-3.400, p = 0.025] for non-diabetics with the Gly/Gly + Gly/Asp genotype, and 3.629 [95% CI = 1.820-7.236, p < 0.001] for diabetics with the Gly/Gly + Gly/Asp genotype. There was no association between Gly1057Asp polymorphism in IRS-2 and HOMA-IR index.

**Conclusion:**

Gly allele at codon 1057 in IRS-2 is correlated with an increased susceptibility to CAD in the Taiwanese population. There is a synergistic effect toward CAD between the pathogenicity of DM and that of the Gly allele.

## Background

There is evidence that coronary artery disease (CAD) is associated with genetic factors [1]. Both insulin resistance [2-4] and diabetes mellitus (DM) [5] are risk factors for CAD. Thus, the genetic variants involved in the development of insulin resistance and DM may be associated with the susceptibility to CAD.

Insulin binding to its receptors leads to activation of several intracellular downstream effectors. Among these, insulin receptor substrate (IRS) proteins are most important [6]. Insulin activates the insulin receptor substrate/phosphatidylinositol 3-kinase (IRS/PI3-K) pathway and the Ras/mitogen-activated protein kinase (MAPK) pathway. The IRS/PI3-K pathway mediates insulin-dependent glucose transport, whereas the Ras/MAPK pathway promotes insulin-mediated cell growth and proliferation. Resistance in the glucose regulation pathway is referred to as insulin resistance. Hyperinsulinemia occurs during insulin resistance due to stimulation of pancreatic β-cell activity. Other pathways are probably over-stimulated due to hyperinsulinemia. The chronic effects of the hyperinsulinemia cause dyslipidemia, higher blood pressure, or platelet hypercoagulation that contribute to CAD pathogenesis [7].

Genetic polymorphism in the IRS plays a role in insulin resistance related disorders [8]. Gly972Arg polymorphism caused by a single nucleotide polymorphism (SNP) in IRS-1 is associated with impaired IRS-1 function [9], reduced insulin sensitivity [10], as well as an increased susceptibility to type 2 DM [11] and CAD [12]. IRS-2 is an alternative effector protein in the insulin signaling pathway [13]. IRS-2 gene knockout mice exhibit a phenotype similar to human type 2 DM [14]. In humans, Gly1057Asp polymorphism in IRS-2 is caused by a SNP in the IRS-2 gene (rs1805097) [15]. Middle-aged glucose-tolerant Danish males who carry the Asp allele have shown decreased serum insulin and C-peptide concentrations during oral glucose tolerance tests [15]. A study of an Italian population reported that the Asp allele increases the risk for type 2 DM in obese subjects, while lean subjects have a decreased risk. Among non-diabetic individuals, fasting C-peptide levels are inversely related to the dosage of the Asp allele [16]. A study of the Chinese Han population also concluded that lean subjects with the Asp allele have a decreased risk for DM, while obese subjects have an increased risk [17]. These data suggest that the Asp allele is associated with a lower risk of insulin resistance and DM in lean individuals. On the contrary, the Gly1057Asp polymorphism in IRS-2 is not associated with insulin resistance in Finnish subjects [18]. The CC genotype of the -756C/T SNP in the promoter region of IRS-2 is associated with an increased susceptibility to CAD [19], which suggests that genetic variants of IRS-2 may be associated with occurrence of CAD. Until now, there are no reports of a relationship between Gly1057Asp polymorphism in IRS-2 and CAD.

The frequency of the Gly allele at codon 1057 in IRS-2 is common in the Chinese Han population [18]. Since most Taiwanese are descendants of Mainland Chinese, they have similar genetic backgrounds. The prevalence of Gly1057Asp polymorphism in IRS-2 is probably common in the Taiwanese. Because CAD shares common risk factors with DM, it is possible that the genetic backgrounds for CAD and DM are similar. Because the Asp allele at codon 1057 in IRS-2 seems to be associated with the low risk of insulin resistance and DM [15,16], we hypothesize that the Asp allele plays a protective role for CAD. In this study, we investigated the relationship between Gly1057Asp polymorphism in IRS-2 and CAD in the Taiwanese population.

## Methods

### Study subjects

A hospital-based cross-sectional study was conducted. Patients older than 18 years old receiving coronary angiography for stable angina pectoris, suspected CAD, or pre-operation examination for structural heart disease were enrolled. We aimed at investigating the association between the Gly1057Asp polymorphism in IRS-2 and coronary atherosclerosis rather than coronary thrombosis; thus, patients with acute myocardial infarction were excluded. Besides, we intended to perform follow-up angiography if indicated; patients with serum creatinine levels greater than 221 μmol/L were excluded to avoid contrast-induced nephropathy. Patient histories of conventional CAD risk factors were obtained. The definitions of hypertension (HTN), type 2 DM, and hypercholesterolemia were in accordance with the relevant reports [20-22]. In brief, a patient was considered to have HTN if a seated blood pressure greater than 140/90 mmHg had been documented on at least two or more office visits, or if antihypertensive medications had been used. Type 2 DM was defined as being present if the patient was under treatment for DM at the time of admission to the study, or if their fasting blood glucose levels had been greater than 7 mmol/L on two or more separate occasions. Hypercholesterolemia was defined as being present if the patient was being treated at the time of enrollment or if their fasting blood total cholesterol levels had been greater than 5.18 mmol/L. Body mass index (BMI) was calculated using the formula: body weight in kilograms divided by the square of body height in meters (kg/m^2^). The homeostasis model assessment of insulin resistance (HOMA-IR) index was calculated in 190 patients of the entire cohort. The HOMA-IR index is a validated tool for estimating insulin resistance, and is calculated as follows: fasting insulin (μIU/ml) × fasting glucose (mmol/ml)/22.5 [23,24]. Blood samples were drawn after at least 8 hours overnight fasting. Fasting plasma sugar was checked by using the standard hexokinase method. Radioimmunoassay was used to check the fasting serum insulin levels. The study protocol conformed to the ethical guidelines of the 1975 Declaration of Helsinki [25] and was approved by the ethics committee of National Cheng Kung University Hospital. After a thorough explanation of the study and its contents, each patient gave their informed consent.

### Detection of Gly1057Asp polymorphism in IRS-2 by polymerase chain reaction (PCR) and restriction fragment length polymorphism (see Additional file 1 and 2)

#### Coronary angiography

Definition of CAD was as described previously [26]. Luminal stenosis was defined as significant when the luminal diameter of the major coronary artery, or one of its major branches, showed a reduction of 50% or greater. Major coronary arteries included left main artery, left anterior descending coronary artery, left circumflex coronary artery, and right coronary artery. The major branches of right coronary artery included posterior descending artery and posterolateral branch, the major branch of left circumflex coronary artery was the major obtuse marginal branch, and the major branch of left anterior descending coronary artery was the major diagonal branch. The decision of coronary arteries with significant luminal stenosis was determined by two independent investigators by visual determination.

### Statistical analysis

The main object of current study is to evaluate the relationship between Gly1057Asp polymorphism in IRS-2 and CAD. Patients without significant luminal stenosis were defined as group A and those with significant stenosis in at least one proper major coronary artery or its major branches were defined as group B. Since the frequency of Gly allele at codon 1057 in IRS-2 in diabetic patients is about 65% for the Chinese Han population [18], and assuming this Gly allele frequency for patients in group B and a 10% difference for patients with the Gly allele in group A, we estimated that 200 patients were needed for each group to provide a two-tailed α of 0.05 and a power of 80%.

For continuous variables, results were presented as mean with standard deviation (SD), and the differences between groups were checked by the independent Student T test. Categorical variables were presented using frequency counts, and intergroup comparisons were analyzed by the chi-square test. A multivariate binary logistic regression analysis was used for CAD prediction. All variables were included in multivariate logistic analysis (enter method). The effect of a risk factor was expressed as an odds ratio (OR) and a 95% confidence interval (95% CI). A multiple linear regression analysis was used to investigate the correlation of variables, including conventional CAD risk factors and genotype, with the HOMA-IR index. Unstandardized regression coefficients (B coefficients) were calculated to show the relationship between the variables and the HOMA-IR index. Standardized regression coefficients (beta coefficients) were also calculated to reveal the effect of each variable on the HOMA-IR index; this allowed the magnitudes of the regression coefficients to be compared and indicated which variable played an important role [27]. The detailed methods were described in Additional file 3 (see Additional file 3).

The concern about multicollinearity in multivariate logistic or multiple linear regression analysis was checked by calculating the variance inflation factor (VIF). Variables with a VIF value greater than 2.5 were considered to have significant multicollinearity with other independent variables. Consequently, any independent variable with a VIF value greater than 2.5 was considered to be dropped from the analysis to avoid multicollinearity, after taking into account the practical aspect and importance of each variable. A p-value of less than 0.05 (two-tailed) was considered significant. All analyses were done using SPSS for WINDOWS, version 12.0 (Chicago, IL, United States).

## Results

### Study subjects

In total, 454 patients were enrolled. The group A contained 170 and the group B contained 284 patients. Table 1 shows the clinical characteristics for the patient cohort. Patients in group B were older. There were significant differences in HTN, DM, hypercholesterolemia history, smoking history, total cholesterol levels, and serum creatinine levels between the two groups. There was a male predilection in group B patients. There were no differences in the BMI between the two groups.

**Table 1:** Demographic characteristics

	**Group A(n** **=** **170)**	**Group B(n** = **284)**	**P value**
Age, years	59.0 ± 13.5	63.6 ± 10.7	<0.001
Body mass index (kg/m^2^)	25.5 ± 4.0	25.7 ± 3.4	0.668
Serum creatinine (μmol/L)	84.8 ± 23.7	94.2 ± 26.0	<0.001
Total cholesterol (mmol/L)	4.8 ± 0.9	5.0 ± 1.0	0.019
Male	82 (48.2%)	192 (67.6%)	<0.001
Hypertension	94 (55.3%)	189 (66.5%)	0.021
Diabetes mellitus	33 (19.4%)	93 (32.7%)	0.002
Hypercholesterolemia history	74 (43.5%)	155 (54.6%)	0.026
Smoking history	43 (25.3%)	114 (40.1%)	0.002

Group A: control subjects.

Group B: CAD patients.

### Frequency of Gly1057Asp polymorphism in IRS-2 in patient cohort

The frequency of Gly allele was 58.6% in total cohort. The frequency of the Gly allele was slightly higher in group B patients than in group A patients but not reached the significant level. Observed genotype frequencies were in Hardy-Weinberg equilibrium (p = 0.884 in all, p = 0.358 in control patients, and p = 0.988 in CAD patients). The frequency of the Gly/Gly and Gly/Asp genotype was significantly higher in group B patients than it was in group A patients (Table 2).

**Table 2:** **Allele and genotype frequency of Gly1057Asp polymorphism of insulin receptor substrate**-**2**

**Allele**	**Allele frequency[number of alleles(%)]**		**P value**
	**Group A (n = 340)**	**Group B (n = 568)**	
Gly	186 (54.7%)	346 (60.9%)	
Asp	154 (45.3%)	222 (39.1%)	0.077
**Genotype**	**Genotype frequency[number of individuals(%)]**		**P value**
	**Group A (n = 170)**	**Group B (n = 284)**	
Gly/Gly + Gly/Asp	126 (74.1%)	241 (84.9%)	
Asp + Asp	44 (25.9%)	43 (15.1%)	0.007

Group A: control subjects.

Group B: CAD patients.

### Relationship between Gly1057Asp polymorphism in IRS-2 and CAD

We analyzed the association between the Gly allele and CAD using binary logistic regression analysis. Besides univariate analysis, all conventional CAD risk factors and genotype were included in multivariate analysis (Table 3). We found that the Gly/Gly and Gly/Asp genotype is a significant risk factor for CAD. Other significant risk factors for CAD were gender, age, DM, and total cholesterol level. The VIF for each variable included in the analysis was less than 2.0.

**Table 3:** **Logistic regression analysis of the relationship between Gly1057Asp polymorphism in IRS**-2 and CAD*

	**Odds ratio(95%CI)(Univariate analysis)**	**P value**	**Odds ratio(95%CI)(Multivariate analysis)**	**P value**
Genotype				
Asp/Asp(n = 87)	reference		reference	
Gly/Gly + Gly/Asp(n = 367)	1.957 (1.220-3.139)	**0**.**005**	2.008 (1.210-3.332)	**0**.**007**
Male	2.240 (1.516-3.309)	<**0**.**001**	1.969 (1.154-3.358)	**0**.**013**
Age, year	1.033 (1.016-1.050)	<**0**.**001**	1.037 (1.017-1.057)	<**0**.**001**
Hypertension	1.609 (1.089-2.376)	**0**.**017**	1.153 (0.738-1.803)	0.531
Diabetes mellitus	2.054 (1.305-3.232)	**0**.**002**	1.847 (1.133-3.009)	**0**.**014**
Smoking history	1.981 (1.302-3.012)	**0**.**001**	1.521 (0.912-2.536)	0.108
Hypercholesterolemia history	1.559 (1.063-2.286)	**0**.**023**	1.308 (0.841-2.032)	0.233
Body mass index(kg/m2)	1.012 (0.960-1.066)	0.667	1.992 (0.936-1.052)	0.796
Total cholesterol(mmol/L)	1.263 (1.037-1.538)	**0**.**020**	1.295 (1.028-1.631)	**0**.**028**
Serum creatitnine(μmol/L)	1.016 (1.007-1.024)	<**0**.**001**	1.004 (0.995-1.014)	0.392

IRS: insulin receptor substrate; CAD: coronary artery disease; CI: confidence interval.

* Totally, 284 CAD patients and 170 control subjects were included in analysis.

### Relationship between the concurrence of Gly1057Asp polymorphism in IRS-2 with DM and CAD

Our multivariate binary logistic regression showed that the association of Gly allele with CAD is independent of the influence of DM. We then evaluated the influence of the concurrence of the Gly allele with DM on the susceptibility to CAD. We stratified the patients who had DM and carried the Gly allele. The odds ratios for patients with CAD were calculated using multivariate binary logistic regression with adjustments for gender, age, HTN, hypercholesterolemia history, smoking history, BMI, total cholesterol and serum creatinine levels. DM was dropped from the analysis to avoid multicollinearity with the variable genotype-DM interaction. Employing non-diabetics with Asp/Asp genotypes as a reference group, the odds ratio for CAD was 1.561 (p = 0.430) for diabetics with Asp/Asp genotypes, 1.922 (p = 0.025) for non-diabetics with Gly/Gly and Gly/Asp genotypes, and 3.629 (p < 0.001) for diabetics with Gly/Gly and Gly/Asp genotypes (Table 4).

**Table 4:** **Logistic regression analysis of the relationship between genotype**-DM interaction and CAD*

	**Odds ratio(95%CI)(Univariate analysis)**	**P value**	**Odds ratio(95%CI)(Multivariate analysis)**	**P value**
Genotype-DM interaction				
Nondiabetics:Asp/Asp(n = 68)	reference		reference	
Diabetics:Asp/Asp(n = 18)	1.768 (0.612-5.105)	0.292	1.561 (0.517-4.713)	0.430
Nondiabetics:Gly/Asp + Gly/Gly(n = 261)	1.782 (1.041-3.050)	**0**.**035**	1.922 (1.086-3.400)	**0**.**025**
Diabetics:Gly/Asp + Gly/Gly(n = 107)	3.505 (1.830-6.711)	<**0**.**001**	3.629 (1.820-7.236)	<**0**.**001**
Male	2.240 (1.516-3.309)	<**0**.**001**	1.981 (1.161-3.378)	**0**.**012**
Age, year	1.033 (1.016-1.050)	<**0**.**001**	1.037 (1.017-1.057)	<**0**.**001**
Hypertension	1.609 (1.089-2.376)	**0**.**017**	1.146 (0.742-1.814)	0.514
DM	2.054 (1.305-3.232)	**0**.**002**	-^#^	-^#^
Body mass index(kg/m^2^)	1.012 (0.960-1.066)	0.667	0.993 (0.937-1.052)	0.802
Hypercholesterolemia history	1.559 (1.063-2.286)	**0**.**023**	1.302 (0.837-2.026)	0.241
Smoking history	1.981 (1.302-3.012)	**0**.**001**	1.516 (0.908-2.530)	0.112
Total cholesterol(mmol/L)	1.263 (1.037-1.538)	**0**.**020**	1.301 (1.033-1.640)	**0**.**026**
Serum creatitnine(μmol/L)	1.016 (1.007-1.024)	<**0**.**001**	1.004 (0.995-1.014)	0.380

DM: diabetes mellitus; CAD: coronary artery disease; CI: confidence interval.

* Totally, 284 CAD patients and 170 control subjects were included in analysis.

^#^ DM was dropped from analysis because the variance inflation factors for DM and genotype-DM interaction were both greater than 10. The variance inflation factor for genotype-DM interaction was less than 2.0 if DM was removed from analysis.

### Relationship between Gly1057Asp polymorphism in IRS-2 and insulin resistance

In 190 of the 454 total study subjects, we investigated the relationship between Gly1057Asp polymorphism in IRS-2 and insulin resistance, which was evaluated using the HOMA-IR index. Among these patients, 82 (43.2%) did not have and 108 (56.8%) had CAD. There were 149 (78.4%) patients with Gly/Gly and Gly/Asp genotypes and 41 (21.6%) patients with Asp/Asp genotypes. Between these two patient groups (Gly/Gly and Gly/Asp genotype *vs*. Asp/Asp genotype), there were no differences in CAD percentage, age, gender, HTN, smoking history, BMI, HOMA-IR index, serum creatinine, or total cholesterol levels. Fifty of 149 (33.6%) patients with Gly/Gly and Gly/Asp genotypes had DM, while 7 of 41 (17.1%) patients with Asp/Asp genotypes had DM (p = 0.041). Using multiple linear regression analysis, all variables including conventional CAD risk factors and genotype were included in analysis to check the relationship between each variable and HOMA-IR index. Employing the Asp/Asp genotypes as a reference, the unstandardized and standardized regression coefficients for Gly/Gly and Gly/Asp genotypes were −0.023 and −0.029 (p = 0.678), respectively (Table 5). These results suggested that there is no relationship between Gly1057Asp polymorphism in IRS-2 and the HOMA-IR index. DM, BMI, and serum creatinine were significantly correlated with the HOMA-IR index. To investigate the influence of obesity on the relationship between Gly1057Asp polymorphism in IRS-2 and insulin resistance, we defined a BMI value greater than 25 kg/m^2^ as obesity according to the Asia-Pacific perspective redefining obesity in adult Asian [28], and repeated multiple linear regression analysis. The unstandardized and standardized regression coefficients for Gly/Gly and Gly/Asp genotypes were −0.028 and −0.035 (p = 0.608), which suggested that there is no relationship between Gly1057Asp polymorphism in IRS-2 and the HOMA-IR index (see Additional file 4). We further performed multiple linear regression for both obese (n = 117) and non-obese (n = 73) groups separately. We did not observe a significant correlation between Gly1057Asp polymorphism in IRS-2 and HOMA-IR index in either group. The VIF for every variable included in each multiple linear regression analysis was less than 2.0.

**Table 5:** **Linear regression analysis for prediction of HOMA**-**IR index**

**Factors**	**Simple regression**		**Multiple regression**		
	**Unstandardizedcoefficient(B)**	**P value**	**Unstandardizedcoefficient(B)**	**Standardizedcoefficient(beta)**	**P value**
Gly1057Asp polymorphism in IRS-2					
Asp/Asp(n = 41)	reference		reference	reference	
Gly/Gly(n = 69) + Gly/Asp(n = 80)	0.007	0.902	−0.023	−0.029	0.678
Age(year)	−0.002	0.245	−0.003	−0.107	0.147
Diabetes mellitus	0.194	<**0**.**001**	0.151	0.213	**0**.**003**
Hypertension	0.103	**0**.**037**	0.078	0.115	0.121
Male	0.019	0.697	−0.032	−0.048	0.574
Smoking	0.013	0.800	−0.012	−0.017	0.830
Total cholesterol(mmol/L)	0.033	0.197	0.040	0.114	0.103
Serum creatinine(μmol/L)	0.002	**0**.**019**	0.003	0.206	**0**.**007**
Body mass index(kg/m^2^)	0.023	<**0**.**001**	0.017	0.189	**0**.**008**

## Discussion

Our study demonstrated an association between Gly1057Asp polymorphism in IRS-2 and CAD. Additionally, we produced evidence that the Gly1057Asp polymorphism in IRS-2 interacts with DM in relation to risk of CAD; there is a synergistic effect toward CAD between pathogenicity of DM and that of the Gly allele.

In our study population, the Gly/Gly and Gly/Asp genotype frequencies for CAD patients were significantly greater than in control subjects (Table 2). These results suggest that the Gly allele positively correlates with the occurrence of CAD, and therefore the Asp allele has a protective effect against CAD. In multivariate binary logistic regression, the odds ratio of CAD was 2.008 for patients with Gly/Gly and Gly/Asp genotypes and 1.847 for those with DM. These results suggest that the Gly1057Asp polymorphism in IRS-2 is independent of DM as a risk factor for CAD and that both Gly allele at codon 1057 in IRS-2 and DM are potent risk factors for CAD (Table 3). Importantly, we found that the concurrence of Gly allele with DM is associated with an increased susceptibility to CAD (Table 4). In patients with either Asp/Asp genotypes or Gly/Gly and Gly/Asp genotypes, the odds ratio for CAD in diabetics is almost twice that of their non-diabetic counterparts, which is compatible with the results reported in the Framingham study [5]. In non-diabetics, the odds ratio for CAD in those with Gly/Gly and Gly/Asp genotypes was 1.922, which was about twice the ratio for those with Asp/Asp genotypes. Similarly, in diabetics, the odds ratio for CAD in those with Gly/Gly and Gly/Asp genotypes was 3.629, which was roughly twice the ratio for patients with Asp/Asp genotypes. Further, the odds ratio for CAD in diabetics with Gly/Gly and Gly/Asp genotypes was 3.629, which was roughly four times the ratio found in non-diabetics with Asp/Asp genotypes (Table 4). These results suggest that the pathogenic ability of the Gly allele to cause CAD is similar to that of DM. They also suggest a synergistic effect between the pathogenicity of DM and that of the Gly allele. We found that there was no correlation between Gly1057Asp polymorphism in IRS-2 and insulin resistance irrespective of BMI values (Table 5) and obesity (Additional file 4). These results probably exclude the possibility that the Gly1057Asp polymorphism in IRS-2 contributes to CAD through altering insulin resistance.

Several studies have already reported that genetic variants caused by a SNP are associated with CAD, including adiponectin receptor 2 gene [29,30], apolipoprotein E gene [31], and genes related to regulation of blood lipid levels [32]. In current study, we found the concurrence of Gly1057Asp polymorphism in IRS-2 with DM affects the susceptibility to CAD. This interaction between Gly1057Asp polymorphism in IRS-2 and DM could be a kind of gene-environmental or gene-gene interaction. DM itself or genes contributory to development of DM could interact Gly1057Asp polymorphism in IRS-2 to influence the susceptibility to CAD. Similar findings for interrelation of genetic variants and DM in relation to disease traits have been reported. Genetic variants of transcriptional factor 7-like 2 gene are associated with CAD, which are significantly modulated by the presence of type 2 DM [33]. Besides, the +183 A/G polymorphism at the 3’-untranslated region of interleukin (IL)-18 genes is associated with the circulating IL-18 levels, especially apparent in patients with type 2 DM and metabolic syndrome [34]. The frequency of Gly allele was 58.6% in our study subjects, which was approximately equal to that found in Chinese and Finnish patients [18]. We found that Gly1057Asp polymorphism in IRS-2 was not associated with insulin resistance, which was consistent with studies of Finnish subjects [18]. However, the Asp allele was reported to be correlated with better insulin sensitivity in specific groups in Danish [15] and Italian [16] populations. This discrepancy may be due to differences in ethnics, or to heterogeneous demographic characteristics. Thus, further investigation is needed to clarify the relationship between Gly1057Asp polymorphism in IRS-2 and insulin resistance.

DM is a potent risk factor for CAD [5]. However, the susceptibility to CAD is different among diabetic patients. In diabetic patients concomitantly with other conventional risk factors for CAD such as hypercholesterolemia and hypertension, the risk for cardiovascular events in the following 5 to 10 years actually increases much more than that for their counterparts [35-37]. Therefore, genetic screening for Gly1057Asp polymorphism in IRS-2 may be a plausible method to early detect diabetic patients with high susceptibilities to CAD.

Our study is a cross-sectional study. The inherent weakness of cross-sectional studies is an inability to indicate causality because the predictor variable is not shown to precede the outcome. However, the predictor variable that we were interested in for this study, Gly1057Asp polymorphism in IRS-2, was determined by the time the study subjects were born. Hence, the results of our study do demonstrate a causal relationship between Gly1057Asp polymorphism in IRS-2 and CAD. Nonetheless, there were several limitations to this study. First, the number of patients for our main objective, investigating the relationship between Gly1057Asp polymorphism in IRS-2 and CAD, was limited. The number of control patients was 170, which is less than the calculated sample size of 200 patients required to achieve a power of 80% with a two-tailed α of 0.05. Thus, a larger scale study is needed to strengthen the present results. The study was also limited in that it was a hospital-based study. There may have been selection bias. Our study subjects were not truly representative of the overall population. Therefore, a general population-based study is needed to confirm our findings. Using multi-slice computed tomography to screen coronary arteries may be a feasible method for this purpose. When we investigated the relationship between Gly1057Asp polymorphism in IRS-2 and insulin resistance we studied only 190 of the total 454 patients. Also, we checked the insulin resistance by calculating the HOMA-IR index rather than by using the gold standard method euglycemic hyperinsulinemic clamp. Hence, our conclusion that there is no correlation between Gly1057Asp polymorphism in IRS-2 and insulin resistance should be further confirmed by an investigation using euglycemic hyperinsulinemic clamp on a larger scale. Finally, although we showed a correlation between Gly1057Asp polymorphism in IRS-2 and CAD, we did not afford the molecular mechanisms underlying this correlation.

## Conclusions

We demonstrated that Gly allele at codon 1057 in IRS-2 is correlated with an increased susceptibility to CAD and that there is a synergistic effect between this polymorphism and DM that increases a person’s susceptibility to CAD. Individual genetic screening for this common polymorphism may facilitate the management of patients, especially for diabetic patients. Further well-designed studies are needed to support our results and to investigate possible clinical and therapeutic applications.

## Abbreviations

BMI: Body mass index; CAD: Coronary artery disease; CI: Confidence interval; DM: Diabetes mellitus; HTN: Hypertension; HOMA-IR: Homeostasis model assessment of insulin resistance; IRS: Insulin receptor substrate; IRS/PI3-K: Insulin receptor substrate/phosphatidylinositol 3-kinase; MAPK: Mitogen-dependent protein kinase; PCR: Polymerase chain reaction; SNP: Single nucleotide polymorphism; VIF: Variance inflation factor.

## Competing interests

The authors declare no potential conflict of interests.

## Authors’ contributions

S-HC conceived this study, coordinated the enrollment of study subjects, superintended assistants to perform laboratory work, and wrote the manuscript. Y-HL and L-MT participated in the coordination of the study and revised the manuscript. J-HC conceived the study and critically revised the manuscript. All authors read and approved the final manuscript.

## Supplementary Material

Additional file 1 **Detection of Gly1057Asp polymorphism in insulin receptor substrate (IRS)-2 by polymerase chain reaction (PCR) and restriction fragment length polymorphism.** We performed genotyping by using PCR and restriction fragment length polymorphism. The detailed methods we used were described. Click here for file

Additional file 2 **Identification of Gly1057Asp polymorphism of insulin receptor substrate (IRS)-2 by polymerase chain reaction (PCR) followed by restriction fragment length polymorphism.** Results of gel analysis for PCR and restriction fragment length polymorphism as well as results of DNA sequencing were shown. Click here for file

Additional file 3 **Statistical analysis strategy for evaluation of relationship between Gly1057Asp polymorphism in IRS-2 and coronary artery disease (CAD) as well as insulin resistance.** Detailed methods for statistical analysis to evaluate relationship between Gly1057Asp polymorphism in IRS-2 and coronary artery disease (CAD) as well as insulin resistance were described. Click here for file

Additional file 4 **Linear regression analysis for prediction of HOMA-IR index.** We performed multiple linear regression analysis to evaluate the relationship between HOMA-IR index and genotype as well as conventional CAD risk factors, including age, gender, diabetes, hypertension, smoking history, total cholesterol level, serum creatinine level, and obesity. There was no relationship between genotype and HOMA-IR index. Diabetes, serum creatinine level, and obesity were significantly correlated to the HOMA-IR index. Click here for file
