# Supplementary material for: Gly1057Asp polymorphism of insulin receptor substrate-2 is associated with coronary artery disease in the Taiwanese population
Source: J Biomed Sci. 2012 Dec 5;19(1):100. doi: 10.1186/1423-0127-19-100 (PMC3541354; doi:10.1186/1423-0127-19-100)
Supplement: Additional file 1 — Detection of Gly1057Asp polymorphism in insulin receptor substrate (IRS)-2 by polymerase chain reaction (PCR) and restriction fragment length polymorphism. We performed genotyping by using PCR and restriction fragment length polymorphism. The detailed methods we used were described. [file 1423-0127-19-100-S1.pdf]

Additional file 1

Detection of Gly1057Asp polymorphism in insulin receptor substrate (IRS)-2 by polymerase chain reaction (PCR) and restriction fragment length polymorphism

Blood was collected in 10 mL Na-EDTA test tubes and stored at -20°C. Genomic DNA was extracted using a commercial kit (QIAGEN) following the manufacturer's recommended procedures and was stored at 4°C in Tris-HCl (10×10^{-3} mol/L) and EDTA (1×10^{-3} mol/L) at pH 8.0 until analysis. DNA extraction and analysis were conducted anonymously and recorded using code numbers.

PCR amplification was conducted in a 50 µL solution containing genomic DNA (500 ng), 20 pmol of each primer, Tris-HCl (10×10^{-3} mol/L, pH 8.3), KCl (50×10^{-3} mol/L), Taq DNA polymerase (1.5 U) (Invitrogen), dNTP (4×10^{-4} mol/L), and $MgCl_2$ (1.5×10^{-3} mol/L). PCR was carried out by denaturation at 94°C for 5 min, and followed by 40 cycles of denaturation at 94°C for 60 s per cycle. Samples were then annealed at 60°C for 60 s. Extension was conducted at 72°C for 60 s, with a final extension at 72°C for 10 min. The forward primer was 5'-TCGCTATTGTCCGCCAGCAG-3'; the reverse primer was 5'-ACACCAAA-AGCCATCTCGGTC-3'. The accession number of the IRS-2 gene sequence is NCBI GenBank : NM_003749, version: 003749.1. A fragment of 398 bp was obtained during the PCR. Restriction enzyme digestion was done at 37°C for 1.5 h in a reaction buffer (15 µL) containing 10 µL of the PCR product, 10X NEB buffer 4 (1.5 µL) (New England Biolabs Inc.), 10X bovine serum albumin (1.5 µL), and restriction enzyme *Hae* II (10 U) (New England Biolabs Inc.). Fragments were then analyzed by 4% agarose gel electrophoresis. Genotypes were determined by two independent investigators who did not know the characteristics of patients. Direct DNA sequencing was performed to confirm the results of PCR and restriction fragment length polymorphism for 3 patients in each genotype. Additional file 2 shows the gel analysis results of Gly1057Asp polymorphism in IRS-2 after PCR and restriction fragment

length polymorphism.
