# Supplementary material for: Gly1057Asp polymorphism of insulin receptor substrate-2 is associated with coronary artery disease in the Taiwanese population
Source: J Biomed Sci. 2012 Dec 5;19(1):100. doi: 10.1186/1423-0127-19-100 (PMC3541354; doi:10.1186/1423-0127-19-100)
Supplement: Additional file 2 — Identification of Gly1057Asp polymorphism of insulin receptor substrate (IRS)-2 by polymerase chain reaction (PCR) followed by restriction fragment length polymorphism. Results of gel analysis for PCR and restriction fragment length polymorphism as well as results of DNA sequencing were shown. [file 1423-0127-19-100-S2.pdf]

Additional file 2

Identification of Gly1057Asp polymorphism of insulin receptor substrate (IRS)-2 by polymerase chain reaction (PCR) followed by restriction fragment length polymorphism

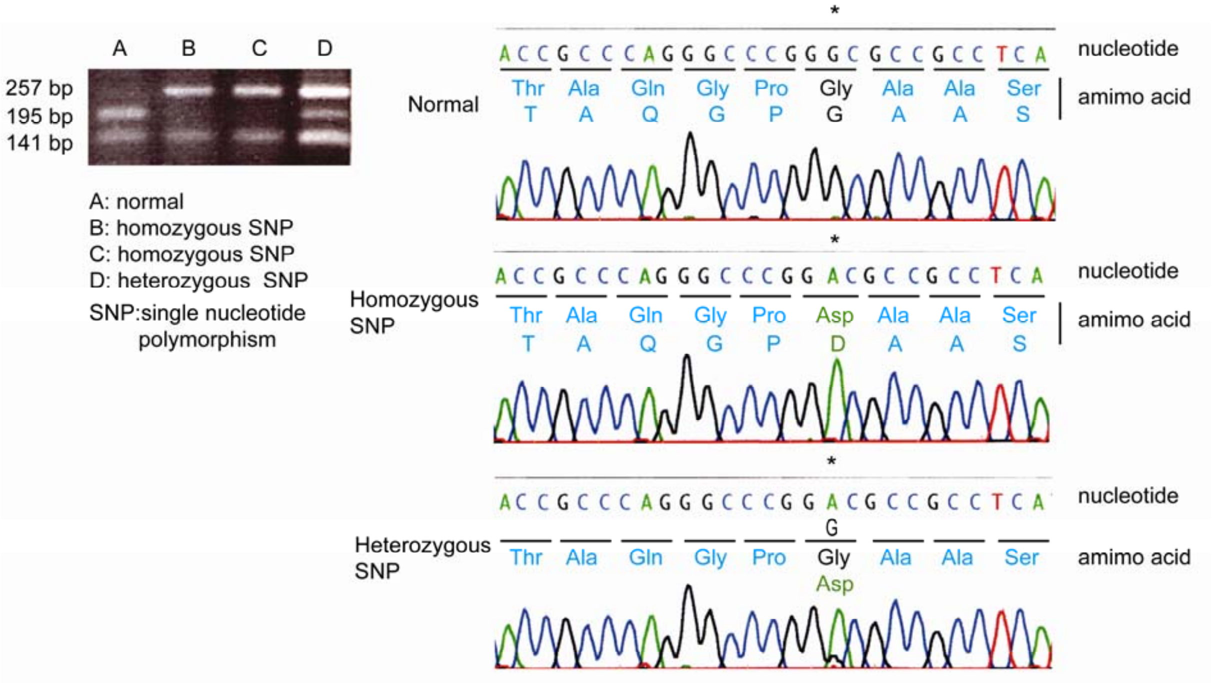

Gly1057Asp polymorphism of IRS-2 was detected using polymerase chain reaction (PCR) followed by restriction enzyme length polymorphism. The PCR product was 398 bp in length. There were two reaction sites for restriction enzyme *Hae* II in normal IRS-2 cDNA. Following *Hae* II digestion, there were 62 bp, 141 bp and 195 bp fragments present. There was only one reaction site for *Hae* II in IRS-2 cDNA bearing single nucleotide polymorphism with substitution of G by A (asterisk) corresponding to Gly1057Asp polymorphism of IRS-2. Following digestion, there were 141 bp and 257 bp fragments present. The products of *Hae* II digestion were analyzed by 4% agarose gel electrophoresis. (Left panel) Lane A is the electrophoresis pattern of normal population (Gly/Gly genotype). Lanes B and C are homozygous single nucleotide polymorphism products (Asp/Asp genotype). Lane D shows the heterozygous polymorphism products (Gly/Asp genotype). (Right panel) The single nucleotide polymorphism of IRS-2 cDNA was confirmed by direct DNA sequencing for 3

patients in each genotype.
