# Supplementary material for: Gly1057Asp polymorphism of insulin receptor substrate-2 is associated with coronary artery disease in the Taiwanese population
Source: J Biomed Sci. 2012 Dec 5;19(1):100. doi: 10.1186/1423-0127-19-100 (PMC3541354; doi:10.1186/1423-0127-19-100)
Supplement: Additional file 3 — Statistical analysis strategy for evaluation of relationship between Gly1057Asp polymorphism in IRS-2 and coronary artery disease (CAD) as well as insulin resistance. Detailed methods for statistical analysis to evaluate relationship between Gly1057Asp polymorphism in IRS-2 and coronary artery disease (CAD) as well as insulin resistance were described. [file 1423-0127-19-100-S3.pdf]

Additional file 3

Statistical analysis strategy for evaluation of relationship between Gly1057Asp polymorphism in IRS-2 and coronary artery disease (CAD) as well as insulin resistance

Statistical analysis strategy for evaluation of relationship between Gly1057Asp polymorphism in IRS-2 and coronary artery disease (CAD)

The frequencies of the Gly1057Asp polymorphism in IRS-2 of the two groups of patients were compared by using the chi-square test. The Hardy-Weinberg equilibrium was tested by the goodness-of-fit chi-square test, to compare the observed genotype frequencies with the expected frequencies. Multivariate binary logistic regression analyses were performed to evaluate the relationship between Gly1057Asp polymorphism in IRS-2 and CAD with adjustments for other conventional CAD risk factors. All variables were included in analysis (enter method). The dependent variable was the presence of CAD. For genotypes, Asp/Asp was coded as zero, while Gly/Gly or Gly/Asp was coded as one. Independent variables included in the analysis were genotype, hypertension (HTN), diabetes mellitus (DM), hypercholesterolemia history, smoking history, gender, age, total cholesterol level, body mass index (BMI), and serum creatinine level. In addition, we investigated the relationship between the concurrence of Gly1057Asp polymorphism in IRS-2 with DM and CAD by using multivariate binary logistic regression analysis with adjustments for conventional coronary risk factors. All variables were included in analysis (enter method). To perform such analysis, we coded non-diabetics with Asp/Asp genotypes as zero, diabetics with Asp/Asp genotypes as one, non-diabetics with Gly/Gly or Gly/Asp genotypes as two, and diabetics with Gly/Gly or Gly/Asp genotypes as three. Independent variables included in the analysis were Gly1057Asp polymorphism in IRS-2 and DM interactions, HTN, hypercholesterolemia history, smoking history, gender, age, total cholesterol level, BMI, and

serum creatinine level. An odds ratio with a 95% confidence interval for the presence of CAD was calculated for each variable.

Statistical analysis strategy for evaluation of relationship between Gly1057Asp polymorphism in IRS-2 and insulin resistance

In 190 patients of the entire cohort, levels of insulin resistance were evaluated using the homeostasis model assessment of insulin resistance (HOMA-IR) index. Multiple linear regression analysis was used to analyze the relationship between Gly1057Asp polymorphism in IRS-2 and HOMA-IR index, with adjustments for other factors. To perform multiple linear regression analysis, categorical variables were recoded as numerical variables. The dependent variable was the HOMA-IR index. For genotypes, Asp/Asp was coded as zero, while Gly/Gly or Gly/Asp was coded as one. Variables included in analysis included genotype, HTN, DM, smoking history, gender, age, total cholesterol level, BMI, and serum creatinine level. HOMA-IR indexes were logarithmically transformed for multiple linear regression analysis. All variables were included in the multiple linear regression. Both unstandardized and standardized regression coefficients were calculated.
