# Supplementary material for: Gly1057Asp polymorphism of insulin receptor substrate-2 is associated with coronary artery disease in the Taiwanese population
Source: J Biomed Sci. 2012 Dec 5;19(1):100. doi: 10.1186/1423-0127-19-100 (PMC3541354; doi:10.1186/1423-0127-19-100)
Supplement: Additional file 4 — Linear regression analysis for prediction of HOMA-IR index. We performed multiple linear regression analysis to evaluate the relationship between HOMA-IR index and genotype as well as conventional CAD risk factors, including age, gender, diabetes, hypertension, smoking history, total cholesterol level, serum creatinine level, and obesity. There was no relationship between genotype and HOMA-IR index. Diabetes, serum creatinine level, and obesity were significantly correlated to the HOMA-IR index. [file 1423-0127-19-100-S4.pdf]

Additional file 4

Linear regression analysis for prediction of HOMA-IR index*

	Simple regression		Multiple regression		
Factors	Unstandardized coefficient (B)	P value	Unstandardized coefficient (B)	Standardized coefficient (beta)	P value
Gly1057Asp polymorphism in IRS-2					
Asp/Asp (n=41)	reference		reference		
Gly/Gly (n=69) + Gly/Asp (n=80)	0.007	0.902	-0.028	-0.035	0.608
Age (year)	-0.002	0.245	-0.003	-0.096	0.195
Diabetes mellitus	0.194	**<0.001**	0.161	0.226	**0.002**
Hypertension	0.103	**0.037**	0.077	0.113	0.129
Male	0.019	0.697	-0.026	-0.039	0.651
Smoking	0.013	0.800	-0.018	-0.024	0.762
Total cholesterol (mmol/L)	0.033	0.197	0.035	0.100	0.155
Serum creatinine (μmol/L)	0.002	**0.019**	0.003	0.195	**0.012**
Obesity (BMI>25 kg/m^2)	0.168	**<0.001**	0.115	0.175	**0.015**

* The homeostasis model assessment of insulin resistance (HOMA-IR) index is an estimate of insulin resistance.
